# β-cyclocitral synergizes the response of adult *Drosophila suzukii* (Diptera: Drosophilidae) to fruit juices and isoamyl acetate in a sex-dependent manner

**DOI:** 10.1038/s41598-019-47081-z

**Published:** 2019-07-22

**Authors:** Jaime C. Piñero, Bruce A. Barrett, Leland Grant Bolton, Peter A. Follett

**Affiliations:** 1 Stockbridge School of Agriculture, University of Massachusetts, Amherst, MA 01003 USA; 2 University of Missouri, Division of Plant Sciences, Columbia, MO 65211, USA; 3 Daniel K. Inouye U.S. Pacific Basin Agricultural Research Center, USDA-ARS, Hilo, HI 96720 USA

**Keywords:** Invasive species, Chemical ecology

## Abstract

Semiochemicals play a pivotal role in the location, evaluation, and utilization of hosts by herbivorous insects. Mixtures of host plant-derived compounds are often required to elicit appropriate levels of response to olfactory stimuli. In multiple-choice bioassays, we characterized the response of adult *Drosophila suzukii* to foliage- and fruit-based synthetic compounds tested alone and in association with grape and tart cherry juices, and assessed whether synergistic interactions among olfactory stimuli are involved in the olfactory-driven behavior of *D. suzukii*. Our results established (1) significant attraction of females (but not males) to β-cyclocitral and isoamyl acetate when tested singly, (2) the presence of a synergistic interaction between β-cyclocitral and cherry juice only for females, and (3) the presence of a synergistic interaction between β-cyclocitral and isoamyl acetate but only in the case of males. Our findings increase our understanding of male and female *D. suzukii* olfactory responses to synthetic compounds and fruit juices as sources of attractants. Combinations of foliage- and fruit-based compounds may be needed to increase SWD attraction.

## Introduction

Chemical cues are considered to play a pivotal role in the location, evaluation, and utilization of hosts by herbivorous insects^1,2^. Numerous studies have shown that, rather than single constituents, mixtures of host plant-derived compounds are often required to elicit appropriate levels of response to olfactory stimuli^3,4^. Furthermore, synergistic interactions among the components of an odor mixture have been found to contribute to the attraction of some species of insects to their host plants^5–7^.

Native to Southeast Asia, the invasive Spotted Wing Drosophila, *Drosophila suzukii* (Matsumura) (Diptera: Drosophilidae)^8,9^, has reached world-wide pest status resulting from its recent invasion of much of North America^10–12^, South America^13,14^ and some regions of Europe^15,16^. Its polyphagous habits, preference for ripening fruits, and the female’s ability to pierce soft-skinned fruits during egg-laying make *D. suzukii* a pest of great economic significance^17,18^. Current management options for *D. suzukii* in fruit crops rely heavily on insecticide applications^19–22^. While early detection and effective monitoring tools are critical to implementation of pest control tactics against *D. suzukii*^23^, effective semiochemically-based monitoring represents a challenge for pest managers given the fly’s ability to reproduce in different host plant species^24,25^, its high reproductive potential^26^, and a general lack of species-specific attractants^23,27^.

While foraging, adult *D. suzukii* seek essential resources suitable for feeding, mating, and oviposition, and volatiles play key roles during this process^28^. Chemical ecology research conducted across the globe has resulted in the identification of volatiles emitted from fermenting materials that are attractive to *D. suzukii*. Synthetic lures have been developed based on blends of four compounds isolated from fermentation headspace of wine and vinegar^29^. The four-component blend has shown to attract more adult *D. suzukii* than apple cider vinegar^29^ and has also shown to detect adults earlier than apple cider vinegar when evaluated in areas that harbor wild blackberry (*Rubus*) species and in commercial blueberry orchards^30^. Given these advantages, commercial food-based lures are currently being used to monitor SWD populations^28,31,32^. However, lures that are based on fermentation materials attract a comparatively high number of other Drosophilid species (and other non-target insects) hindering trap performance and increasing sorting time^26,31,33^.

A promising alternative to the use of fermentation-derived attractants is the identification of volatiles emitted by foliage and/or host fruits. Keesey *et al*.^34^ reported a positive behavioral response of male and female *D. suzukii* towards the strawberry leaf terpenoid β-cyclocitral. Similar results were found under laboratory conditions when β-cyclocitral was evaluated in 60 ml plastic containers. EAG recordings demonstrated that the antennal response of *D. suzukii* to β-cyclocitral is species-specific, given the lack of EAG detection to the same compound by *D. melanogaster* and *D. biarmipes*^34^.

Attraction of adult *D. suzukii* to fruit volatiles emitted by intact blackberry (*Rubus fruticosus* L), blueberry (*Vaccinium corymbosum* L.), cherry (*Prunus cerasus* L.), raspberry (*Rubus idaeus* L.), and strawberry (*Fragaria ananassa* Duchesne) has been reported by Revadi *et al*.^35^. Further evidence that fruit volatiles are important in *D. suzukii* host location was provided by Abraham *et al*.^36^, who documented fly attraction to fruit extracts of highbush blueberry, cherry, raspberry, and strawberry, and to selected volatile compounds derived from raspberry fruit. Using behavioral and electrophysiological assays, Revadi *et al*.^35^ found that isoamyl acetate, a fruit-based compound, elicited a positive behavioral response in *D. suzukii*^35^. This compound also attracts other Drosophilids^37^. More recently, a quaternary blend made of ethyl octanoate, acetoin, acetic acid, and ethyl acetate, compounds present in headspace volatiles collected from fermented apple juice, was reported to be 2–4 times more attractive and 2–3 times more selective than the widely used ACV and commercially available SWD lures under field conditions^38^.

To date, the role of synthetic or natural sources of plant-based odor in the olfactory response of male and female *D. suzukii* is little understood. The main goals of this study were (1) to quantify the response of male and female *D. suzukii* to the leaf compound β-cyclocitral and to the fruit compound isoamyl acetate tested singly against mineral oil (solvent control), and tested against each other, (2) to assess the types of interactions between β-cyclocitral and isoamyl acetate when tested alone or in combination, and (3) to assess whether β-cyclocitral and isoamyl acetate would increase the response of adult *D. suzukii* to grape and tart cherry juices.

## Results

### Experiment 1

**Figure 1:**
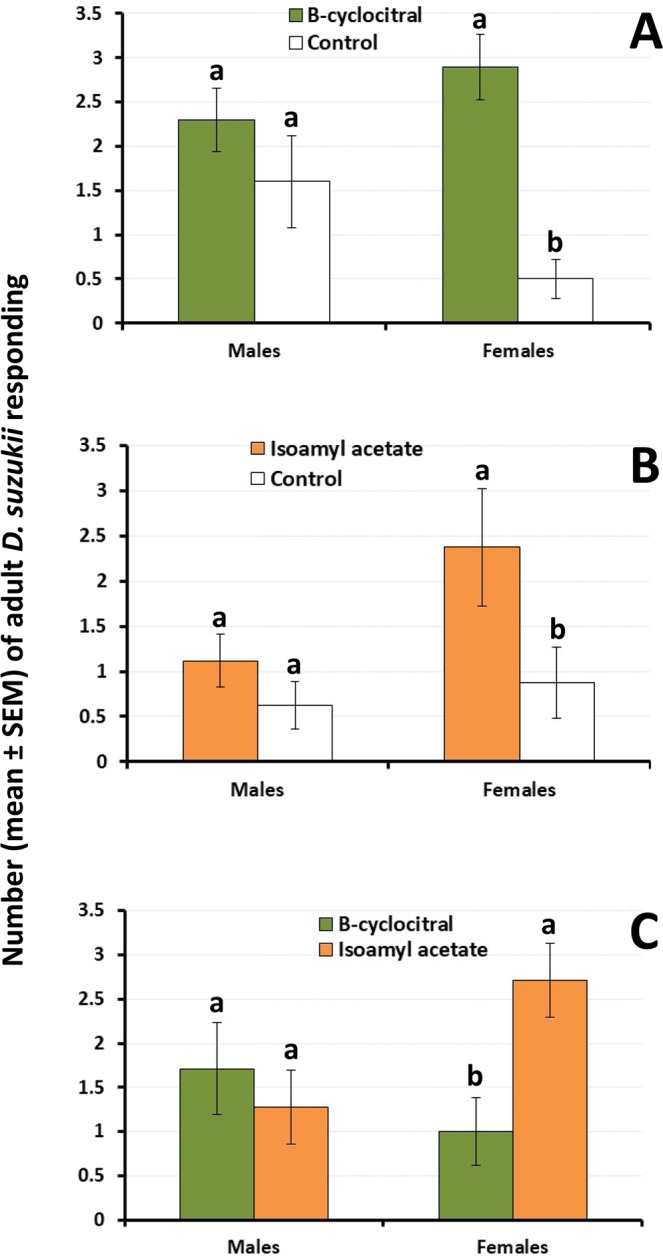
Response of male and female *D. suzukii* to (**A**) β-cyclocitral and (**B**) isoamyl acetate when compared singly against mineral oil (control), and to (C) β-cyclocitral and isoamyl acetate, tested against each other. For each sex, different letters above bars denote significant differences according to t-tests at P = 0.05.

Significant interactions between main effects (sex and treatment) were detected for the comparison of (1) β-cyclocitral against control (mineral oil) and (2) β-cyclocitral against isoamyl acetate, but not for the comparison of isoamyl acetate against control (Table 1). When tested singly, β-cyclocitral and isoamyl acetate were not attractive to *D. suzukii* males, compared to mineral oil (t-tests t_18_ = 1.4; P = 0.167 and t_14_ = 1.2; P = 0.236 for β-cyclocitral and isoamyl acetate, respectively) (Fig. 1A,B). In contrast, both chemical compounds were attractive to females (t-tests t_18_ = 4.1; P < 0.001 and t_14_ = 2.2; P = 0.043 for β-cyclocitral and isoamyl acetate, respectively) (Fig. 1A,B). When tested singly against each other, β-cyclocitral and isoamyl acetate were similarly attractive to males (t-test t_12_ = 1.7; P = 0.126) and isoamyl acetate was significantly more attractive to females than β-cyclocitral (t-test t_12_ = 3.0; P = 0.010) (Fig. 1C).

**Table 1:** Statistical output for the main and interactive effects of ‘olfactory treatment’ and ‘sex’ on the level of adult *Drosophila suzukii* response to plant-derived volatiles inside cages.

Experiment	Variables	DF	*F*	P
**1** (β-cyclocitral against control)	Treatment	1	16.55	0.001
	Sex	1	1.48	0.232
	Treatment x sex	1	4.92	0.033
	Error	36		
**1** (isoamyl acetate against control)	Treatment	1	6.35	0.018
	Sex	1	1.48	0.121
	Treatment x sex	1	4.92	0.298
	Error	28		
**1** (β-cyclocitral against isoamyl acetate)	Treatment	1	4.37	0.047
	Sex	1	0.24	0.622
	Treatment x sex	1	11.81	0.002
	Error	24		
**2** (β-cyclocitral alone and in combination with isoamyl acetate)	Treatment	3	5.45	0.001
	Sex	1	0.25	0.618
	Treatment x sex	3	1.21	0.308
	Error	184		
**3** (grape juice and β-cyclocitral)	Treatment	3	21.52	<0.001
	Sex	1	0.02	0.886
	Treatment x sex	3	0.50	0.268
	Error	56		
**3** (grape juice and isoamyl acetate)	Treatment	3	11.76	<0.001
	Sex	1	1.61	0.211
	Treatment x sex	3	0.89	0.455
	Error	44		
**3** (cherry juice and β-cyclocitral)	Treatment	3	13.22	<0.001
	Sex	1	5.74	0.019
	Treatment x sex	3	4.21	0.009
	Error	64		
**3** (cherry juice and isoamyl acetate)	Treatment	3	9.07	<0.001
	Sex	1	9.59	0.003
	Treatment x sex	3	3.40	0.024
	Error	52

### Experiment 2

**Figure 2:**
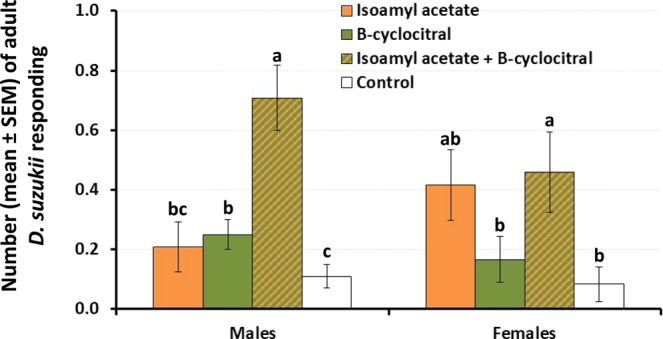
Response of male and female *D. suzukii* to β-cyclocitral and isoamyl acetate either, alone or in combination, and to mineral oil (control). For each sex, different letters above bars denote significant differences according to ANOVA and Fisher-protected LSD tests at P = 0.05.

A 2-way ANOVA detected a significant effect of “treatment” and “sex” on the response of *D. suzukii* to the treatments evaluated, and the interaction term was non- significant (Table 1). A significant effect of olfactory treatment was detected for both males (ANOVA F_3,92_ = 3.4; P = 0.021) and females (ANOVA F_3,92_ = 3.2; P = 0.025). While the level of response of males to isoamyl acetate or β -cyclocitral alone did not differ significantly from the response to mineral oil, and synergistic effect was noted when both compounds were presented in combination (Fig. 2). Females showed a significant preference for isoamyl acetate over β-cyclocitral, and the level of response to the combination of isoamyl acetate and β-cyclocitral did not differ from the response exhibited to isoamyl acetate alone (Fig. 2).

### Experiment 3

#### Male and female *D. suzukii* response to synthetic compounds in association with grape juice

**Figure 3:**
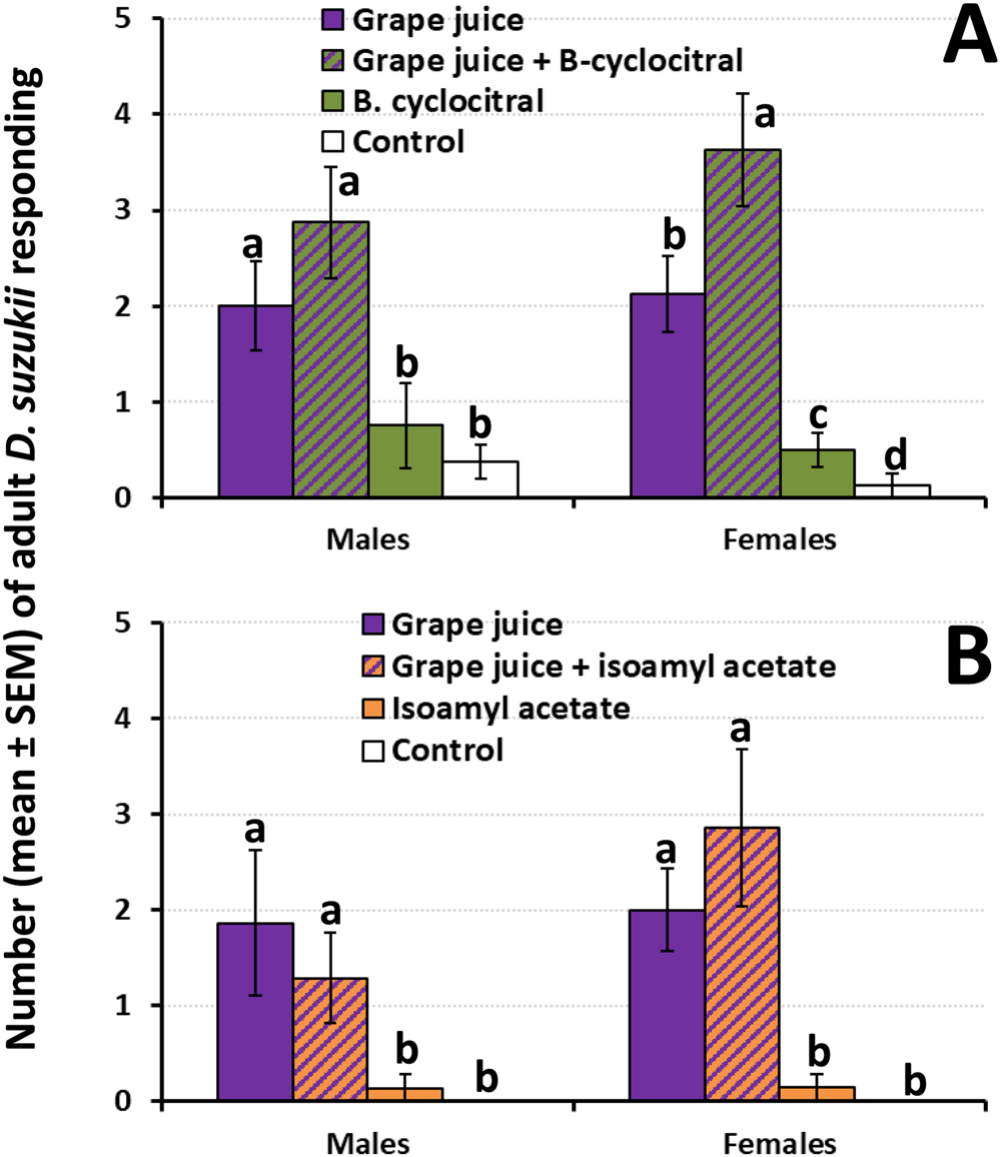
Response of male and female *D. suzukii* to Concord grape juice either, alone or in association with (**A**) β-cyclocitral, and (**B**) isoamyl acetate. Mineral oil was used as control. For each sex, different letters above bars denote significant differences according to ANOVA and Fisher-protected LSD tests at P = 0.05.

For both β-cyclocitral and isoamyl acetate the outcomes of the 2-way ANOVAS revealed a significant effect of treatment, a non-significant effect of sex, and non-significant interaction terms (Table 1). Grape juice was attractive to males (ANOVA F_3,28_ = 6.8; P < 0.01) and the level of response was not significantly enhanced by the addition of β-cyclocitral (Fig. 3A). For females, grape juice alone was attractive and the addition of β-cyclocitral to grape juice significantly increased the response (ANOVA F_3,28_ = 16.8; P < 0.001) compared to grape juice alone. The effect was additive (Table 2). β-cyclocitral was attractive to females, but not to males, when compared against water (Fig. 3A).

**Table 2:** For experiment 4, type of interactions between single synthetic compounds (b-cyclocitral, isoamyl acetate), fruit juices (cherry, grape) and combined attractants as determined by Ratios of Interaction (ROI)^45,46^.

Fruit juice	Gender	Plant volatile	Ratio of Interaction (ROI)^a^	Type of interaction
Grape	Male	β-cyclocitral	1.14 ± 0.30	Additive
Grape	Female	β-cyclocitral	1.86 ± 0.61	Additive
Grape	Male	Isoamyl acetate	0.82 ± 0.40	Additive
Grape	Female	Isoamyl acetate	1.37 ± 0.30	Additive
Cherry	Male	β-cyclocitral	1.00 ± 0.41	Additive
Cherry	Female	β-cyclocitral	3.85 ± 0.81*	Synergistic
Cherry	Male	Isoamyl acetate	0.70 ± 0.37	Additive
Cherry	Female	Isoamyl acetate	1.58 ± 0.37	Additive

^a^Mean ± SE; *P < 0.05 for the null hypothesis that ratio of interaction (ROI) = 1 by a two-tailed *t* test.

For the tests that involved isoamyl acetate, grape juice alone was attractive both to males (ANOVA F_3,22_ = 3.4; P = 0.04) and females (ANOVA F_3,22_ = 10.0; P < 0.001) when compared to the control (Fig. 3B). The addition of isoamyl acetate to grape juice did not increase significantly the level of response of males or females compared to grape juice alone. Isoamyl acetate was not attractive to males or females (Fig. 3B).

#### Male and female *D. suzukii* response to synthetic compounds in association with cherry juice

**Figure 4:**
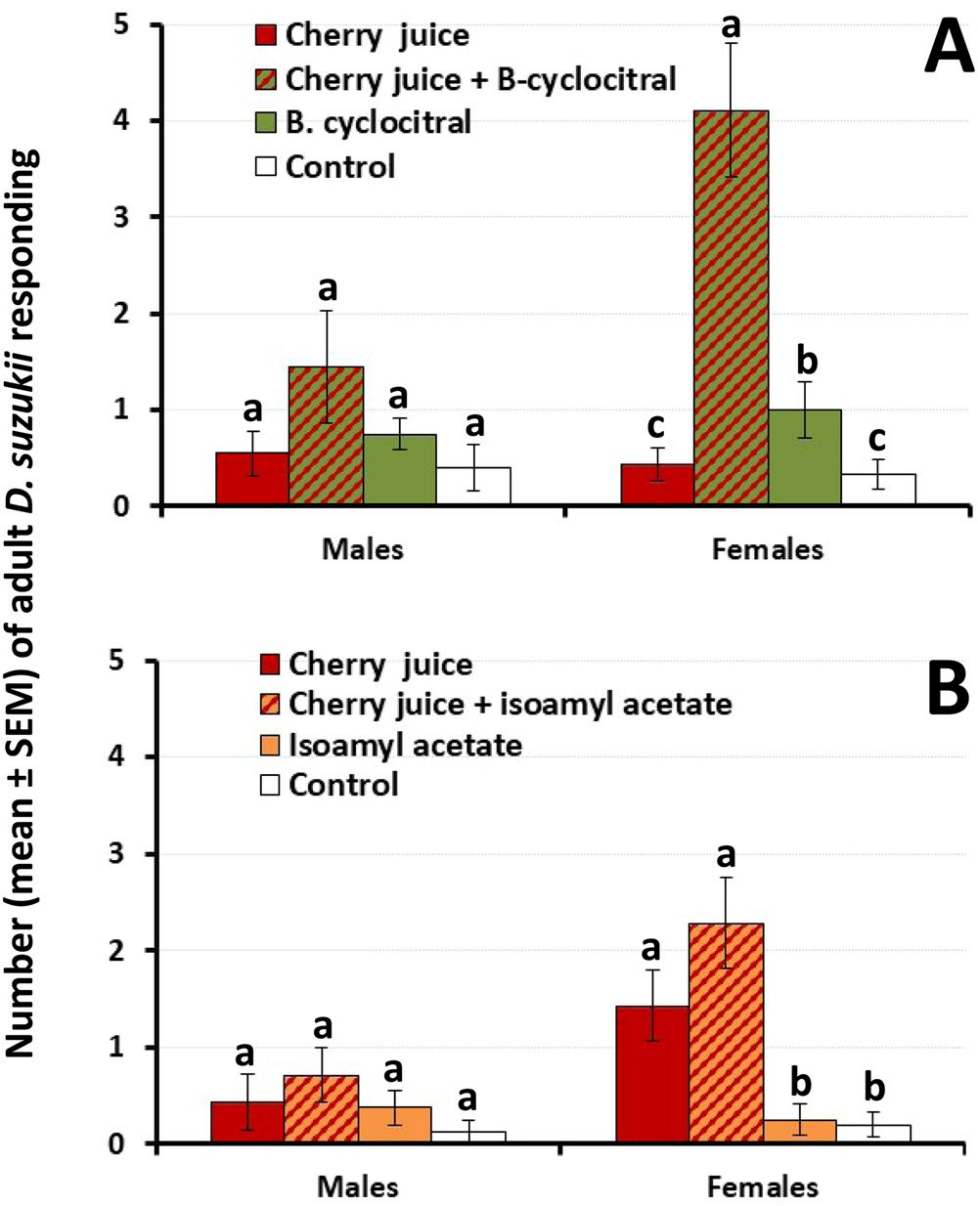
Response of male and female *D. suzukii* to tart cherry juice either, alone or in association with (**A**) β-cyclocitral, and (**B**) isoamyl acetate. Mineral oil was used as control. For each sex, different letters above bars denote significant differences according to ANOVA and Fisher-protected LSD tests at P = 0.05.

The outcomes of the 2-way ANOVAS revealed a significant effect of treatment and sex (main factors) and a significant treatment x sex interaction (Table 1) indicating dissimilar responses by males and females to the olfactory treatments when cherry juice was included in the evaluations. For males, no significant preference for cherry juice either, alone or in combination with β-cyclocitral, or for β-cyclocitral alone was recorded (ANOVA F_3,32_ = 1.2; P = 0.320). For females, cherry juice and β-cyclocitral interacted synergistically (Table 2), resulting in significantly increased attraction (ANOVA F_3,32_ = 17.4; P < 0.001) (Fig. 4A). β-cyclocitral was attractive to females when compared to the control.

Cherry juice and isoamyl acetate either, alone or in combination, were not attractive to males (ANOVA F_3,26_ = 1.2; P = 0.343). In contrast, female *D. suzukii* showed a significant preference for cherry juice and for cherry juice + isoamyl acetate when compared to isoamyl acetate alone and the control (ANOVA F_3,26_ = 10.2; P < 0.001) (Fig. 4B).

## Discussion

The results of the behavioral experiments demonstrated: (1) positive responses of *D. suzukii* females (but not males) to isoamyl acetate and β-cyclocitral when presented singly, (2) a significant preference of females (but not males) for isoamyl acetate over β-cyclocitral, (3) a significant increase in the level of response of females to grape juice when β-cyclocitral was added and a female-specific synergistic interaction between β-cyclocitral and cherry juice, and (4) a male-specific synergistic interaction between β-cyclocitral and isoamyl acetate when these compounds were presented in association.

*Drosophila suzukii* is known to colonize host plants in the early stages of fruit ripening. Therefore, it is reasonable to hypothesize that adults use chemical cues emitted from both foliage and fruit during host location^28,36^. The synthetic compounds evaluated in this study, β-cyclocitral and isoamyl acetate, are present in various host^34,35^ and non-host plants (e.g., Saglam *et al*.^39^) of *D. suzukii*, and they were selected based on reported positive physiological and behavioral responses of adult *D. suzukii* (e.g.^34,35^).

The results from the first experiment involving synthetic compounds indicated positive responses of *D. suzukii* females (but not males) to β-cyclocitral and to isoamyl acetate when tested singly against the solvent mineral oil. Previously, Keesey *et al*.^34^ demonstrated that both male and female *D. suzukii* are sensitive physiologically and respond behaviorally to this compound. In turn, isoamyl acetate is present in the aroma of many fruit species and it was found in the headspace of the yeast *Hanseniospora uvanum*^40^, a species found in the alimentary canal of adult *D. suzukii*^41^. In the present study, isoamyl acetate was significantly more attractive to females than β-cyclocitral when these compounds were tested against each other. Females used in all experiments were sexually mature and presumably mated therefore those individuals were considered to have a high oviposition drive. Males, however, did not discriminate between the leaf and fruit compounds a result that suggests that both types of compounds may play a role in male attraction to host plants. Keesey *et al*.^34^ hypothesized that β-cyclocitral is used as a possible long-range cue in selectively attracting *D. suzukii* to the vicinity of a fruiting plants. The synergistic effect of β-cyclocitral and isoamyl acetate noted for males, but not for females, suggests that both compounds may be involved in host and mate finding and courtship behaviors^28^. Whether age or mating status influence the response of males and females to β-cyclocitral remains to be determined.

Isoamyl acetate did not increase significantly the response of males and females to either fruit juice. It is conceivable that this result may be due to redundancy, as isoamyl acetate was found to be present in several commercial fruit juices (J.C. Piñero *et al*. unpub. data). However, when β-cyclocitral was added to cherry juice it synergistically enhanced the response of females to the binary combination, a result that suggests that both foliage and fruit compounds may be needed to elicit high levels of response in females seeking for food or oviposition resources.

A variety of insect herbivores are indeed attracted to specific sorts of volatile chemicals, usually in an appropriate blend, emanating from their respective hosts. Within the sensory modality of olfaction, a quantitative dependent synergism between the fermentation materials acetic acid and acetoin in the response of *D. suzukii* was documented by Cha *et al*.^33^. Similar results were seen with the closely related *D. melanogaster*^42^, suggesting that the synergistic relationship between acetic acid and acetoin may be conserved in the melanogaster group. Synergistic interactions among components of host-plant odor have been reported in the Oriental fruit moth, *Grapholita molesta* (Busk)^7^ and in the grapevine moth, *Lobesia botrana* (Denis & Schiffermüller)^43^ (both Tortricidae). For the former species, Piñero and Dorn^7^ identified a bioactive 5-compound mixture composed of two constituents, green leaf volatiles and aromatic compounds, and reported that benzaldehyde and benzonitrile must be present in association with three distinct green leaf volatiles to produce an attractant effect on *G. molesta* females. In turn, Tasin *et al*.^43^ reported a strong synergistic effect of a blend of the grape volatiles β-caryophyllene, (E)-β-farnesene and (E)-4,8-dimethyl-1,3,7-nonatrieneon on female *L. botrana* attraction, and omission of any one compound from this 3-component blend almost abolished attraction.

Collectively, our findings (1) increase our understanding of male and female *D. suzukii* foraging behavior in response to synthetic compounds and natural juices as potential sources of attractants, and (2) support the conclusion that olfactory synergisms play a role in host finding behavior in *D. suzukii*, and such effects seem to apply differently to males and females. Improved efficacy of *D. suzukii* kairomonal lures is desirable to improve monitoring systems and potentially for the development of more effective semiochemically-based management.

## Methods

### Behavioral bioassays

All studies were conducted from 28 June to 7 August 2017 at the University of Hawaii Agricultural Experiment Station in Kainaliu, Hawaii Island, and from 10 May to 15 July 2018 at the University of Massachusetts, Amherst, Massachusetts. All experiments were conducted using screened cages (60 cm^3^) positioned inside a shade house (Kainaliu) and in the laboratory (Amherst). Four equidistant wires (15 cm in length) were suspended at each of the four corners of the cages, to hang the sources of volatiles.

#### Insects

Adult *D. suzukii* stemmed from colonies established at the Daniel K. Inouye U.S. Pacific Basin Agricultural Research Center, United States Department of Agriculture (USDA) Agricultural Research Service (ARS), Hilo, Hawaii Island, and at the University of Massachusetts (Amherst) campus. Fruit fly rearing followed procedures described by Follett *et al*.^44^. In short, immature stages were reared in 275-ml plastic containers with ventilated lids on diet that consisted of agar (13.5 g), water (1,500 ml), cornmeal (37.5 g), sugar (60 g), nutritional yeast (21 g), methyl paraben (1 g, dissolved in 10 ml of 90% ethanol), and propionic acid (12 ml). Rearing conditions were 24 °C (±2 °C) and a photoperiod of 12:12 (L:D) h. Upon adult emergence, adults were transferred to adult cages as needed to maintain a relatively constant adult density. Flies were provided water (one cup) and a 20% sugar/water solution (one cup) ad libitum. All flies were tested when they were 2–3 d old.

#### Sources of volatiles

The synthetic compounds tested were β-cyclocitral (≥95%) and isoamyl acetate (≥95%) (Sigma-Aldrich Chemical Co., Milwaukee, WI). β-cyclocitral is a volatile isoprenoid present in green plant foliage including strawberry^34^, a host plant of *D. suzukii*. Isoamyl acetate is a compound present in ripening fruit^35^. Each compound was evaluated at 10^−2^ concentration based on Bolton *et al*. (unpub. data). Concord grape and tart cherry juices (Knudsen Just Juice, Knudsen & Sons, Inc., Chico, CA) were purchased from a local store.

For each experiment, 200 µL of each material (either, a particular synthetic compound at the 10^−2^ concentration in mineral oil, mineral oil alone, or undiluted fruit juice) were pipetted onto clear 1.5 ml centrifuge tubes. Prior to treatment application, the lids of the centrifuge tubes were removed, a 3 cm wire was wrapped around their neck, and a thin coating of Tangletrap insect coating (Tanglefoot Company, Grand Rapids, MI) was applied to the outer surface of the tubes to capture alighting flies. For all experiments, Tangletrap-coated tubes were placed in all four corners of the cage.

For experiment 1, which compared the flies’ response to two olfactory treatments (synthetic compound versus mineral oil as control), two centrifuge tubes loaded with a particular compound were hung from each of the two diagonally opposite corners in the cage and the other two corners received mineral oil. For experiments 2, 3, and 4, which involved evaluations of materials tested singly or in combination, two centrifuge tubes were fastened together using wire. One tube received a particular juice type (or synthetic compound), and the second tube was loaded with a particular synthetic compound. To account for the additional surface area, for the treatments that received only one treatment (either, a synthetic compound, a particular fruit juice, or the solvent mineral oil), a second, empty Tangletrap-coated tube was attached to the treated tube. Under this approach, each corner of the cage had two centrifuge tubes.

#### Experimental approach

For experiments 1, 3 and 4, observations took place between 7:00 a.m. and 12:00 p.m. in shade house (Kainaliu, HI). For experiment 2, trials were conducted from 8:00 to 12:00 pm in the laboratory (Amherst, MA). The ranges of temperature and relative humidity values during the observations were 20–26 °C and 65–85%, respectively for Kainaliu (HI), and 21–24 °C and 65–75%, respectively, for Amherst. For each trial, 10 males and 10 females were introduced into each cage using two glass vials (diam: 1.5 cm; height: 7 cm). The lid was removed to allow adult *D. suzukii* to disperse within the cage and acclimatize to cage conditions. Observations started about 20 min after fly release, as soon as the olfactory treatments were prepared and deployed. Each trial lasted 4 hours, time during which the number of males and females responding (i.e., landing on a centrifuge tube) was recorded. All responders were removed using an aspirator.

#### Experiment 1

This series of three tests evaluated the response of male and female *D. suzukii* to (1) β-cyclocitral and (2) isoamyl acetate, tested singly against mineral oil (solvent control) and (3) β-cyclocitral versus isoamyl acetate. Trials were replicated 10–11 times.

#### Experiment 2

This experiment examined the type of interactions existing between β-cyclocitral and isoamyl acetate in terms of attractiveness to male and female *D. suzukii*. The olfactory treatments that were evaluated were: (1) isoamyl acetate alone, (2) β-cyclocitral alone, (3) isoamyl acetate in association with β-cyclocitral, and (4) mineral oil (control). Trials were replicated 24 times.

#### Experiment 3

For this set of evaluations, we aimed at identifying the type of interactions existing between β-cyclocitral and isoamyl acetate and two types of fruit juices. More specifically, we quantified the response of male and females to (1) Concord grape juice, either alone or in combination with β-cyclocitral versus β-cyclocitral alone and mineral oil (control), and (2) grape juice, either alone or in combination with isoamyl acetate versus isoamyl acetate alone and mineral oil. The aforementioned approach was repeated in a separate set of trials using tart cherry juice instead of grape juice. Each trial was replicated 8 times.

### Data analyses

For all experiments, the number of adult *D. suzukii* responding to the olfactory treatments over the 4-hour period were compared by two-way Analysis of Variance (ANOVA) with “treatment” and “sex” as main factors. Because a significant interaction was detected in 4/8 analyses (see Results section), we then subsequently compared, for each sex, the level of male and female response to the olfactory treatments using t-tests (Experiment 1) and one-way ANOVAS (Experiments 2 and 3). Data were transformed using √(x + 0.5) prior to analysis to stabilize variances. Means were separated, whenever appropriate, by a Fisher’s Least Significant Differences test at the 5% probability level. In addition, for experiment 3 we performed comparisons of ratios of interaction (ROIs)^45,46^ to examine type of interactions (additive or synergistic) among single (a particular plant volatile, a particular fruit juice) vs. 2-component odor treatments. In our case, a ROI = [(A + B) + control]/[(A) + (B)], where (A) represents adult SWD response to a particular plant volatile alone, (B) is the response of adults to a particular fruit juice alone, (A + B) denotes attraction to the combination of a particular plant volatile and a particular fruit juice, and control represents SWD responses recorded in vials having mineral oil. ROI values equal to 1 indicate additive effects. ROI values significantly greater than 1 indicate synergistic interactions between chemicals, and values significantly less than 1 denote inhibitory^45,46^. Analyses were done in STATISTICA 13^47^. All figures show untransformed data.
